# Memristive Characteristics of the Single-Layer P-Type CuAlO_2_ and N-Type ZnO Memristors

**DOI:** 10.3390/ma15103637

**Published:** 2022-05-19

**Authors:** Wenqing Song, Xinmiao Li, Ruihua Fang, Lei Zhang

**Affiliations:** 1 State Key Laboratory of High Performance Complex Manufacturing, College of Mechanical and Electrical Engineering, Central South University, Changsha 410083, China; sevtar@163.com (W.S.); lixinmiao95@csu.edu.cn (X.L.); ruihuagua@foxmail.com (R.F.); 2 School of Intelligent Manufacturing and Mechanical Engineering, Hunan Institute of Technology, Hengyang 421002, China

**Keywords:** ZnO, NiO, memristors, single-layer, oxygen ion migration

## Abstract

Memristive behaviors are demonstrated in the single-layer oxide-based devices. The conduction states can be continually modulated with different pulses or voltage sweeps. Here, the p-CuAlO_2_- and n-ZnO-based memristors show the opposite bias polarity dependence with the help of tip electrode. It is well known that the conductivity of p-type and n-type semiconductor materials has the opposite oxygen concentration dependence. Thus, the memristive behaviors may attribute to the oxygen ion migration in the dielectric layers for the single-layer oxide based memristors. Further, based on the redox, the model of compressing dielectric layer thickness has been proposed to explain the memristive behavior.

## 1. Introduction

As the fourth fundamental electronic element, a memristor was theoretically conceived through symmetry arguments by Leon Chua in 1971 [1]. The first physical memristor was fabricated in 2008 using the combination of different-oxygen-content TiO_2_ bi-layer structures [2], and the compressing insulator layer thickness model was proposed based on the migration of the oxygen ion. The memristor has potential application for the nonvolatile memories, neural network simulation, and even new and unexplored functionalities in electronics [3], thus the memristor has received wide attention. Several groups have succeeded in designing and fabricating memristors using different materials and structures [4,5,6,7,8,9] to realize the inherent learning and memory functions. Among the memristors, the single-dielectric layer memristor has been used to emulate human memory behaviors and realize the transition from short-term memory to long-term memory [9]. On the other hand, memristive behaviors seem to be related to the migration/diffusion of the oxygen ion for oxide-based memristors. It is well known that the p-type and n-type semiconductor materials have the opposite oxygen concentration dependence. Based on the redox, some opposing resistive switching behaviors usually are observed in p-type and n-type oxides, such as the bias polarity dependence and the forming location of the conductive filaments [10,11,12]. This may be attributed to the different conductive nature for p-type semiconductor (cation vacancy) and n-type semiconductor (oxygen vacancy) [13,14]. Our pre-study indicates that CuAlO_2_ is a p-type semiconductor, and its resistance value will gradually decrease with increasing oxygen concentration of the CuAlO_2_ film [12,13,14,15,16]. However, for the n-type materials (e.g., ZnO), the resistance value will increase gradually with the increasing oxygen concentration of the film [17,18]. Comparing to their memristive behaviors may be an indirect but effective way to reveal the memristive mechanism for the p-type and n-type single-layer oxide memristors.

In this paper, the single-layer Pt/p-CuAlO_2_/Pt and Pt/n-ZnO/Pt devices show the memristive behaviors. A model of compressing dielectric layer thickness is proposed to explain the memristive characteristics, and the effect of oxygen on single-layer memristors has been also studied.

## 2. Experimental 

**Figure 1:**
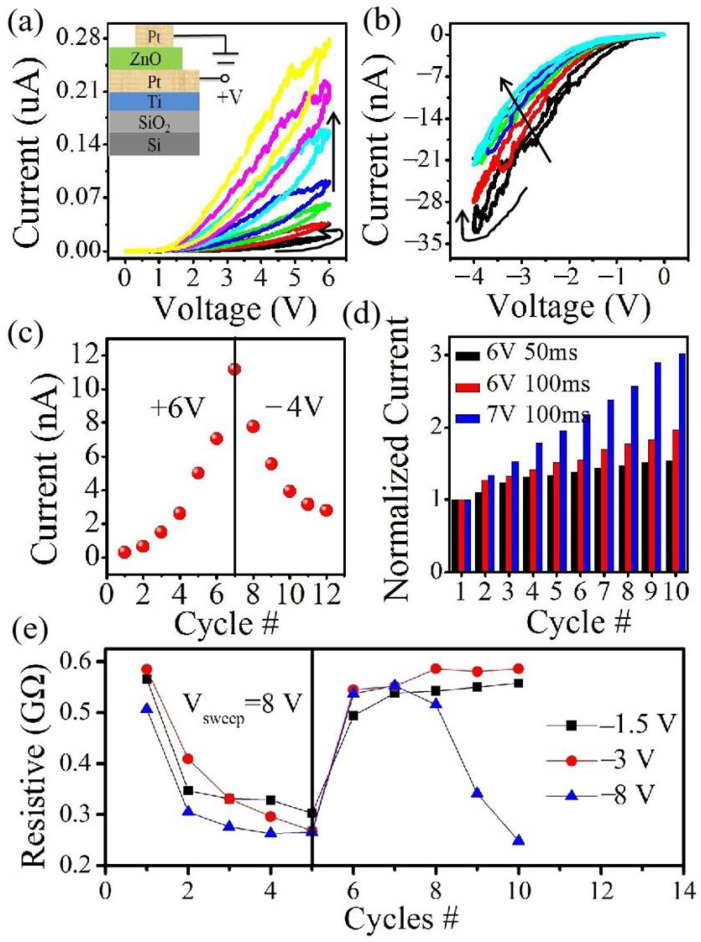
I-V characteristics of the Pt/ZnO/Pt device (inset) at (**a**) positive and (**b**) negative bias voltages. The device conductivity continuously increases (decreases) during the positive (negative) voltage sweeps. (**c**) Variation of the device conductivity with the scanning cycles. (**d**) Change of device conductivity with the consecutive pulses. Higher-amplitude and longer-duration pulses cause a larger change in the device conductivity. (**e**) Gradual variation of the resistance with positive and negative double voltage sweeps. The different amplitudes of the negative voltages present the different final resistance states.

**Figure 2:**
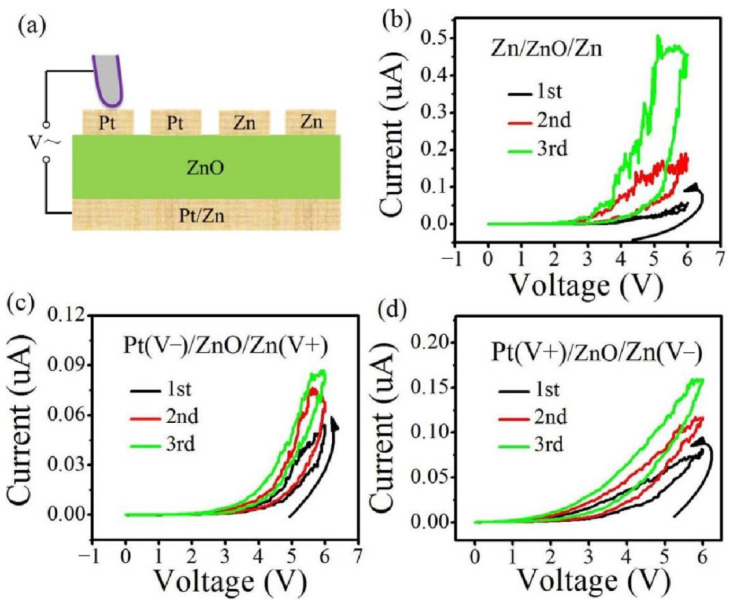
(**a**) Schematic diagram of ZnO-based single-layer memristors with different electrodes; (**b**) Zn/ZnO/Zn device; (**c**) Pt/ZnO/Zn device with the negative voltage adding to the Pt electrode; (**d**) Pt/ZnO/Zn device with the positive voltage adding to the Pt electrode. The conductivity of these devices gradually increases during the continuous positive voltage sweeps, and all of the devices show similar memristive behaviors.

**Figure 3:**
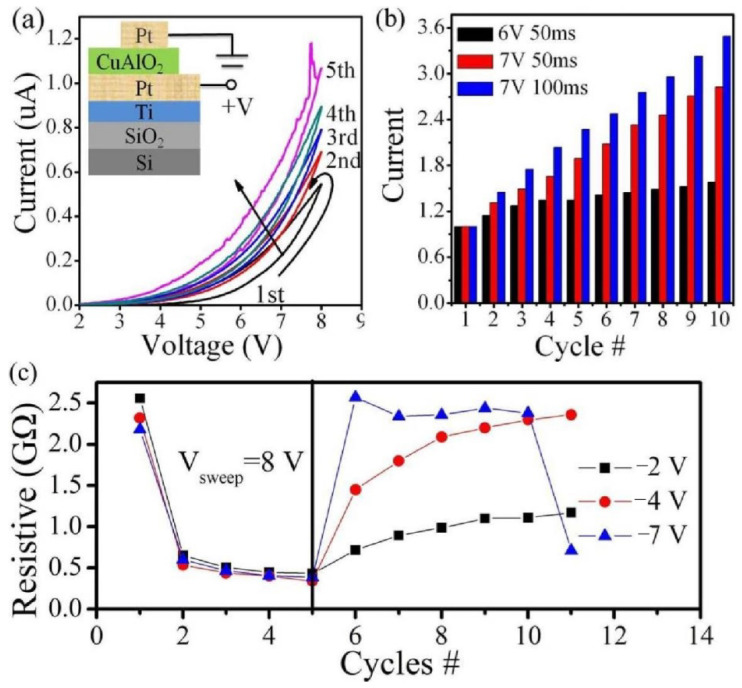
(**a**) The gradual increase of the current with positive double voltage sweeps for the Pt/CuAlO_2_/Pt device (inset). (**b**) The change of device conductivity with the consecutive pulses. Higher-amplitude and longer-duration pulses cause a larger change in the device conductivity. (**c**) The gradual variation of the resistance with positive and negative double voltage sweeps. The different amplitude of the negative voltages will present the different final resistance states.

The structural diagrams of ZnO memristor devices are shown in the inset of Figure 1a. About 60 nm-thick n-ZnO film was deposited on Pt/Ti/SiO_2_/Si substrates (Pt bottom electrode (BE)) by magnetron sputter, and the growth was performed in 2 Pa O_2_ atmosphere at room temperature. Then, the Pt top electrode, which was patterned into many circular pads with a 500 μm diameter using a shadow mask, was thermally evaporated on the top to complete the fabrication of Pt/n-ZnO/Pt memory cell. Meanwhile, the Zn/ZnO/Zn and Pt/ZnO/Zn reference devices were also fabricated in the Figure 2a. We also fabricated the Pt/~60 nm p-CuAlO_2_/Pt device; the growth of CuAlO_2_ film was performed in 2 Pa O_2_ atmosphere at room temperature in the inset of Figure 3a. To exploring the memristive mechanism, the n-ZnO/Pt and p-CuAlO_2_/Pt device without the Pt top electrode were fabricated, and the Pt probe was applied to complete the electrical measurement. Here, the Pt probe was grounded, and the voltage source was applied to the Pt bottom electrode. 

## 3. Results and Discussion

A primary criterion to evaluate the memristive behaviors is the pinched voltage-dependent current behaviors [19]. When consecutive double voltage sweeps are applied to the Pt/n-ZnO/Pt device, its conductivity continuously increases. Then the conductivity continuously decreases when negative voltage sweeps are applied to the device, as shown in Figure 1a,b. Such a changing trend of the current is plotted in Figure 1c. It can be seen that the resistance continuously decreases with the positive voltage sweeps, while the resistance continuously increases with reverse voltage sweeps. In addition, we studied the effect of the negative voltage on the memristive behaviors in Figure 1e. The resistance values were read at the end of each sweep. When the consecutive positive (0 to 8 V) voltage sweeps are applied to the Pt/ZnO/Pt device, the resistance values continuously decrease from the initial state resistance to relative low resistance states. Once the negative voltage sweeps are applied from 0 to −1.5 V, the resistance values continuously increase. Following, the consecutive positive voltage sweeps from 0 to 8 V and negative voltage sweeps from 0 to −3 V were applied to the same device. The resistance value recovers faster to the initial value with the bigger negative voltage. Once increasing the negative voltage sweeps to −8 V after the consecutive positive voltage sweeps, the resistance increases to the initial value right away. Then the resistance values re-decrease with the sweep cycles for the single-layer memristor devices, as discussed below. The device conductivity can also be adjusted by tuning the duration and amplitude of the applied voltage pulses. It was found that the higher-amplitude and longer-duration pulses cause a larger change in the conductivity, as shown in Figure 1d. The memristive behaviors have also been tested for these memristor devices with different electrodes, such as Pt/ZnO/Zn and Zn/ZnO/Zn devices. When the consecutive voltage sweeps regardless of the direction are applied to all the devices, the current continuously increases and the resistance continuously decreases. 

All the single-layer memristor devices with different electrodes (Figure 2a) show normal memristor behaviors, as shown in Figure 2b–d. It indicates that the memristive behaviors are independent of the electrode material. In addition, the effect of the thickness of ZnO film on the memristive behavior has been also studied. The memristive behavior will disappear once the thickness of ZnO layer is above 300 nm (not shown), which illustrates that the strong electric field is necessary to realize the memristive characteristics for the single-layer oxide-based memristors.

Figure 3 shows the memristive characteristics of Pt/p-CuAlO_2_/Pt device. The positive voltages and pulses induce the continuous increase in current (Figure 3a,b, respectively). Meanwhile, higher-amplitude and longer-duration pulses cause a larger change in the conductivity. We also observed that the negative voltage can effectively modulate the resistance value in Figure 3c. The resistance value shows a similar resistance changing trend with the single-layer Pt/n-ZnO/Pt memristor device (Figure 1e). It seems to show similar memristive characteristics to the single-layer Pt/n-ZnO/Pt and Pt/p-CuAlO_2_/Pt devices. 

**Figure 4:**
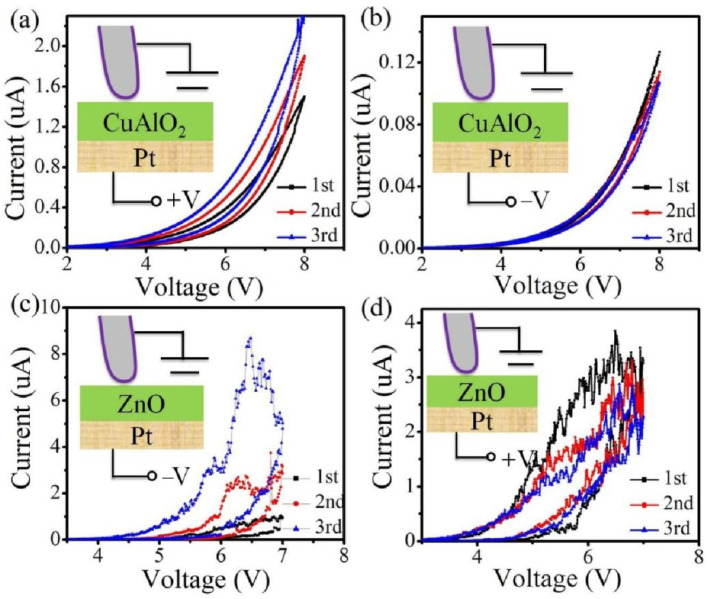
The memristive behaviors are measured by the Pt tip electrode. (**a**,**b**) I-V characteristics of the CuAlO_2_/Pt device with positive and negative voltage sweeps. (**c**,**d**) I-V characteristics of the ZnO/Pt device with negative and positive voltage sweeps.

To explore the memristive mechanism, n-ZnO/Pt and p-CuAlO_2_/Pt devices were fabricated. The Pt probe was applied to record the change of the resistance states. Figure 4a,b shows the I–V curves of CuAlO_2_/Pt device with the applied continuous positive and negative voltage sweeps, respectively. When continuous positive voltage sweeps are applied to the BE, the current gradually increases, while the current is nearly invariant with continuous negative voltage sweeps. For the ZnO/Pt device, the continuous negative voltage sweeps make the current gradually increase, while the current is nearly invariant with the continuous positive voltage sweeps (Figure 4c,d). We note that when either the positive or negative voltage is applied to the devices in Figure 1a or Figure 3a, the memristive behaviors (the variation of resistance states) will appear. The different memristive phenomenon may be understood that the Pt electrode can effectively prevent the oxygen fleeing from the medium layer. Compared with Figure 4a,c, the opposite bias polarity is required to realize the memristive behaviors for the p-type CuAlO_2_ and n-type ZnO memristor devices. It is well known that the resistance states of the n-type and p-type oxides semiconductor have the opposite oxygen concentration dependence. Thus, the oxygen ions migration in the films under the field are important to realize the memristive behaviors.

**Figure 5:**
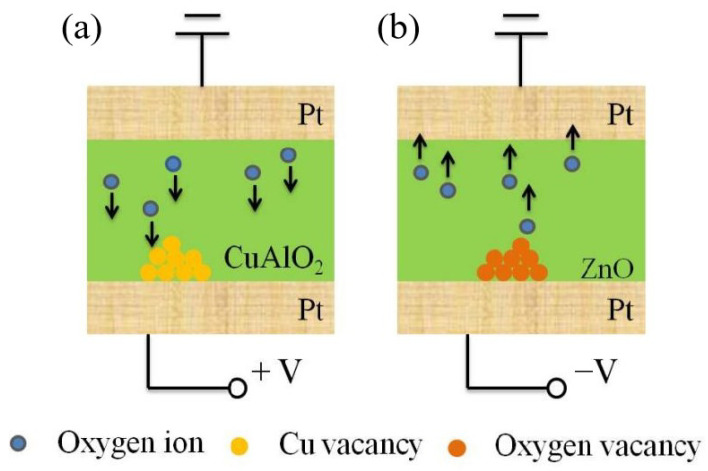
The schematic of the memristive behaviors for (**a**) Pt/CuAlO_2_/Pt and (**b**) Pt/ZnO/Pt memristor devices.

Since Williams et al. proposed ion migration as the basis of memristive operation [2], several memristive models have been built utilizing silver or oxygen ions migration. Such an oxygen ion migration operation in the films is important to realize our single-layer oxide-based memristive behaviors. Thus, the redox is expected to explain the changing of resistance states; meanwhile, the memristive model of compressing insulator layer thickness is further proposed for single-layer oxide-based Pt/p-CuAlO_2_/Pt and Pt/n-ZnO/Pt memristors. The initial resistance of both devices show a relative high resistance value. Our pre-study proved that CuAlO_2_ is a p-type semiconductor [12]. The excess oxygen can increase the number of cation vacancies (e.g., Cu vacancies), enhancing the conductivity of p-type CuAlO_2_ film. When a positive voltage is applied to the Pt/p-CuAlO_2_/Pt device, oxygen ions will move to the BE side, enhancing the concentration of Cu vacancies. Therefore, Cu vacancies will accumulate and compress the thickness of the insulator layer, leading the resistance to gradually decrease, as shown in Figure 5a. On the other hand, it is well known that ZnO is a typical n-type semiconductor, and the deficient oxygen state can increase the concentration of oxygen vacancy, enhancing the device conductivity. Thus, a negative voltage will increase the number of oxygen vacancies near the BE for the Pt/n-ZnO/Pt device, and compress the thickness of the insulator layer, decreasing the device resistance, as shown in Figure 5b. 

Based on the memristive model of compressing insulator layer thickness, some phenomena have been well explained. The accumulation of the Cu vacancies and oxygen vacancies will gradually decrease the resistance value of Pt/CuAlO_2_/Pt and Pt/ZnO/Pt memristors with the voltage sweeps, respectively. When the small opposite voltages are applied to both devices, the accumulation of Cu vacancies or oxygen vacancies will continuously decrease, leading to a gradual increase in the resistance of the devices. Once the big opposite voltages are applied, the accumulated Cu vacancies or oxygen vacancies will disappear right away. Meanwhile, the accumulation of the Cu vacancies or oxygen vacancies will appear at the other Pt electrode. Thus, the accumulation leads to an increase in the device resistance and then a decrease with the big opposite voltage sweeps. In addition, for the Pt probe measurement, once a negative voltage is applied to the p-CuAlO_2_/Pt device, oxygen ions in the CuAlO_2_ film will move to the Pt probe and enter into the atmosphere. The Cu vacancies of the CuAlO_2_ film will not accumulate near the Pt probe, thus the memristive behavior will disappear. For the n-ZnO/Pt device, a positive voltage leads to oxygen ions moving to the BE. The oxygen vacancies are difficult to accumulate near the Pt probe, thus the memristive behavior will also disappear. Based on the redox model, an opposite bias polarity will be required to induce the same memristive behaviors for the single-layer p-CuAlO_2_ and n-ZnO memristor devices. Meanwhile, a detailed comparison between previously reported memristors and our single-layer memristor is provided in Table 1. It is clear that our memristor device has the simplest structure. More importantly, the memristive mechanism has been studied by the probe measurement.

## 4. Conclusions

To conclude, the single-layer oxide-based memristor devices are demonstrated, and oxygen ion migration operation has been proposed. The memristive behaviors of Pt/p-CuAlO_2_/Pt and Pt/n-ZnO/Pt devices are attributed to the accumulation of Cu vacancies and oxygen vacancies near the electrode, and compressing the thickness of insulator layer, respectively. Therefore, the opposite bias polarity is required to induce p-type CuAlO_2_ and n-type ZnO into the same resistance state transformation, reflecting their carrier types.

## Figures and Tables

**Table 1:** A comparison with previous literature.

Device Structure	Memristive Behaviors	Mechanism Research	Memristive Model
Ag/CH_3_NH_3_PbI_3_(OHP)/ITO [6]	Yes	No	No
ITO/Nb-doped SrTiO_3_ heterojunction [5]	Yes	No	No
Ni/p-NiO/N-ZnO/Ni [8]	Yes	No	Yes
Pt/CuAlO_2_/Pt or Pt/ZnO/Pt (our work)	Yes	Yes	Yes

## Data Availability

The data presented in this study are available on request from the corresponding authors.
